# The Flexibility of Fertility Preferences in a Context of Uncertainty

**DOI:** 10.1111/padr.12114

**Published:** 2017-12-20

**Authors:** Jenny Trinitapoli, Sara Yeatman

From Ryder's (1973) “cloudy future” to Lee's (1980) “moving target,” the malleable nature of fertility preferences is widely accepted; however, tools for conceptualizing and measuring preferences have been slow to evolve. In this article, we seek to demonstrate that underpinning the *number* people give when asked about their ideal family size, and critical to interpreting it, is either a rigidness or a flexibility that is contextually situated and dynamic over the life course. To illustrate the difference between fixed and flexible orientations to fertility, we draw upon the metaphor of a movable feast. While some religious holidays like Christmas and All Souls Day occur annually on fixed days, others like Passover, Easter, and Pentecost change from year to year depending upon the lunar cycle or other ecclesiastical dates. From the perspective of the Gregorian calendar, movable feasts seem irregular, unpredictable, and sometimes merely seasonal. But movable feasts are no less regular than fixed feasts, nor are they of secondary importance; they differ in being governed by a distinct, flexible logic. Like many major religious feasts, some people's fertility preferences are indeed fixed, but it is the movable ones we endeavor to theorize here.

Our interest in flexibility is anchored in two recent empirical and theoretical developments in the demographic literature. First, although instability in fertility preferences is higher in developing contexts than in the West (Bankole and Westoff 1995; Kodzi, Johnson, and Casterline 2010), new evidence suggests that this preference instability is not simply random noise but frequently patterned. In Malawi, for example, the setting of our present study, women change their numeric and timing preferences in response to changes in their relationships (divorce, widowhood, new marriage) and reproductive circumstances (pregnancies and child mortality) (Sennott and Yeatman 2012; Yeatman, Sennott, and Culpepper 2013). Furthermore, instability itself has predictive power with respect to short‐term fertility outcomes (Kodzi et al. 2010).

Second, scholars have developed more theoretically sophisticated ways of thinking about fertility. The Theory of Conjunctural Action (TCA) (Johnson‐Hanks et al. 2011) critiques theories of fertility that reduce it to planned action because they rely on assumptions of clarity about a predictable, imagined future. Such an idealized view of reality is difficult or impossible to reconcile with the messiness of real lives, in which young adults lack “definite knowledge about their future family formation, education, and career paths, and so are likely to form their fertility intentions based on general social norms rather than specific desires” (Hayford 2009: 767). Fertility preferences, therefore, should only rarely be treated as a fixed statement of a feasible plan, and researchers should expect fertility behaviors to respond to contingencies, inputs, and shifts that occur at the micro and macro levels. Despite widespread agreement on these points, how can demographers go about integrating notions of flexibility into empirical research on fertility?

## Uncertainty and fertility in Africa

Our interest in the nature of flexibility is further motivated by the puzzle of fertility transition in sub‐Saharan Africa. The shape of the African fertility transition is distinct from the patterns of decline that characterized Latin America and Asia during the second half of the twentieth century (Bongaarts and Casterline 2013; Casterline and El‐Zeini 2007). The debate about whether Africa's fertility transition is late, stalled, or simply different is ongoing (Bongaarts 2017; Bongaarts and Casterline 2013; Caldwell and Caldwell 2002; Caldwell, Orubuloye, and Caldwell 1992; Casterline and Agyei‐Mensah 2017; Mbacké 1994; Moultrie, Sayi, and Timæus 2012; Shapiro and Gebreselassie 2008; Smith 2004). Despite differing in their views of the nature of the transition, scholars agree that fertility preferences play a central role in the transition and in our capacity to develop a better understanding of it. Fertility preferences are critical because, in the words of Bongaarts and Casterline (2013: 159), they “represent a key link in the chain of causation between fertility and its socioeconomic determinants.”

Questions about the nature of fertility preferences raise especially challenging issues for researchers working in sub‐Saharan contexts. While some researchers maintain that fertility rates in sub‐Saharan Africa remain high precisely because desired fertility has remained high (Bongaarts 2006; Bongaarts and Casterline 2013; Pritchett 1994), others read the evidence differently. Günther and Harttgen (2016), for example, document that across the region realized fertility has exceeded wanted fertility by two children for more than two decades. They interpret this gap as evidence that African women are less capable of translating child preferences into outcomes than are women in other developing contexts. An extensive literature on fertility in sub‐Saharan Africa attributes the observed gaps between preferences and completed fertility to “unmet contraceptive need” (Bankole and Ezeh 1999; Bongaarts 1991; Frank and Bongaarts 1991; Sedgh and Hussain 2014). Complicating this interpretation are three factors: i) the important but often neglected caution against inferring individual intentions from population rates (Johnson‐Hanks 2007); ii) the weak relationship between family planning programs and fertility reduction (Günther and Harttgen 2016); and iii) new evidence that unrealized fertility is far more prevalent in this region than previously recognized. Recent estimates from individual‐level analyses reveal that despite high levels of fertility in sub‐Saharan Africa, as many as 46 percent of African women fall short of their ideal at the end of childbearing (Casterline and Han 2017).

Other common explanations for the fact that preferences and behaviors tend to be misaligned in sub‐Saharan Africa include poor data quality (Dare and Cleland 1994), poor construct validity (Bankole 1995), and the possibility that reproductive decisions remain outside the calculus of conscious choice (Coale 1973; van de Walle 1992). To readers familiar with recent developments in the methods and materials of demography, the limits of these explanations are apparent. While concerns about data quality are valid, data availability (if not quality) has been improving, and statistical methods for treating preferences as dynamic processes have advanced considerably. Non‐numeric responses to questions about fertility desires such as “Don't know” and “Up to God” have declined (Frye and Bachan 2017), suggesting that the vast majority of women think numerically about the future with respect to their families.

## Uncertainty and strategic flexibility

Whereas the presence of some uncertainty about one's future is universal, most high‐fertility societies are characterized by rampant uncertainty, and scholars from various disciplines have argued that flexibility is a strategic response to the many uncertainties of life in the African context. The connection between uncertainty and flexibility has been elaborated by scholars of rural livelihoods working among Kenyan pastoralists (Butt 2011), Yoruba cacao farmers (Berry 1993), and Mandara Mountain dwellers (Lev and Campbell 1987). In settings where “no condition is permanent” (Berry 1993), flexibility in everything from the selection of land and crops to the timing of labor for planting and harvest is crucial to survival in both the short and long term. Similarly, flexibility in childbearing is a strategic response to life's uncertainties.

Describing the landscape of her research site, Johnson‐Hanks (2005: 364) underscored the exceptionally high levels of existential and economic uncertainty. “Life in contemporary Cameroon is extremely uncertain, both in the specific sense that death often comes early and unexpectedly and also more generally: few events in everyday experience are predictable or consistent. From buses to paychecks to roadblocks to prices, common things elude standardization.” Where day‐to‐day life is riddled with uncertainties, being flexible about all sorts of things—including childbearing (how much and when)—is a necessity (Johnson‐Hanks 2005). Posited as an alternative to rational‐choice perspectives on action, the notion of “judicious opportunism” captures the ease with which individuals can withdraw from their prior intentions (intentions that were real when articulated) by seizing opportunities to reach desirable ends rather than struggling against tides to manifest a fixed and actionable plan. According to Johnson‐Hanks, judicious opportunism is found not just in Cameroon but wherever the usual supports for rational choice are wobbly. And while the flexibility that judicious opportunism requires may look, on the surface, like “just waiting” or like indecision or inaction, flexibility is distinct from these responses in that it is strategic.

Examples of uncertainty and its relationship to fertility abound; here we point to two additional examples—one emphasizing the existential and the other the economic. Extending the literature on insurance or replacement effects on fertility (Cain 1981; Caldwell et al. 1992; LeGrand et al. 2003; Randall and LeGrand 2003), Sandberg (2006) used network data from an agrarian community in Nepal to demonstrate that uncertainty about child survival (proxied by high levels of infant mortality within a network of conversational partners) accelerated and increased women's fertility. Using qualitative data from peri‐urban Mozambique, Agadjanian (2005) looked not to the graveyard but to the market, arguing that stated fertility desires are conditional on current economic and social circumstances and that reproductive aspirations (especially at lower parities) should be treated as tentative because they are shaped by assessments of an unknowable future. While Agadjanian acknowledged the persistent poverty that characterizes much of his study population, he emphasized not the poverty itself but “the unpredictability of the economic situation” (2005: 625), including structural factors like labor market opportunities and intimate conjunctures like spousal migration and relationship strain.

Because the data demands for examining preference change are high, most evidence comes from the data‐rich West (Hayford 2009; Heiland, Prskawetz, and Sanderson 2008; Iacovou and Tavares 2011; Liefbroer 2009; Udry 1983); however, the evidentiary basis from Africa is growing (e.g., Kodzi et al. 2010; Yeatman et al. 2013). The roots of this literature on preference change can be found in theories about the impact of child mortality, post‐hoc rationalization, and household bargaining, but new research shows that other types of events provide change as well. In our context of Malawi, a wide array of conjunctures is known to affect both the number of children a woman wants and will subsequently have and the timing of those pregnancies and births. Confirmed or suspected HIV infection, for example, leads women to accelerate their childbearing plans in order to achieve their ideal family size while still in good health (Trinitapoli and Yeatman 2011), while caregiving responsibilities for non‐biological children (fostering) often lead women to reduce their numeric preferences and delay their childbearing (Bachan 2015). Labor market opportunities may lead women to adjust their timing preferences (Sennott and Yeatman 2012), while expectations that any serious relationship would be solidified through offspring mean that partnership changes in the wake of death or divorce tend to increase desired fertility (Verheijen 2013; Yeatman et al. 2013).

In sum, fertility preferences are contingent and are unstable over time in ways that are patterned. What remains less clear is whether and to what extent strategic flexibility i) varies with perceptions and experiences of uncertainty, ii) may help account for the high levels of preference instability observed in sub‐Saharan Africa, and iii) helps explain a unique pattern of fertility‐related behaviors and outcomes.

## Previous efforts to measure preference flexibility

Reflecting on his experience directing the US National Fertility Study (NFS), Ryder likened the task of asking American respondents to identify their optimal reproductive target to “asking the respondent to perform a complex conceptual experiment: ‘If everything else in your life were to remain the same, except for your parity, what would you choose for your parity?’” He continued, “I suspect that respondents, faced with this challenge, can scarcely avoid thinking of other things they would like to change in addition to the number of children, such as their health, or their housing, or perhaps their husband, unless, of course, they reject the game altogether and converge on their actual experience” (Ryder 1973: 504). When researchers ask questions such as “If you could have exactly the number of children you want, what number would that be?,” women almost always answer clearly, providing a single number. However, underpinning these numbers are processes that are messy to model but central to understanding fertility preferences and what they do (and do not) tell us.

During the heyday of research on fertility preferences, several scholars sought to supplement best‐practice measures of ideal family size (IFS) with new constructs that could tap, prospectively, an underlying structure that would tell us more. For example, Coombs advanced both theory and measurement related to fertility preferences, employing the metaphor of preferences unfolding around a personal ideal (the target). She codified this metaphor in a set of preference scales that were part of a broader endeavor to generate more valid, sensitive, and refined measures of preferences: “[I]f we are to explore in more precise fashion than heretofore the antecedents and correlates of preferences, measures beyond global statements about preferred numbers provide valuable tools” (Coombs 1974: 609). Coombs‐authored preference scales force respondents to move a beyond their initial target to reveal underlying preferences. To describe her “unfolding theory” of fertility, Coombs began with an exercise that moved respondents to either end of a constrained spectrum of ideal family size: “‘If you couldn't have ___ (number given) would you rather have __ (lower number) or __ (higher number)?’ and so on until the respondent chose zero or six” (1974: 588–89). Today, the most widespread adaptation of the Coombs scale forces respondents to choose second and third preferences, which enables analysts to identify women's underlying preference for a small or a large family but masks variability in movement up and down the IFS spectrum by constraining the amount of variation within each sample to two shifts per person.

Concerned primarily with the instability of fertility preferences over the life course, Morgan (1981) built upon Coombs's insights, offering a simple but elegant alternative. Leveraging changes in preferences observed among American women from the National Fertility Studies conducted in 1965 and 1970, he insisted: “This uncertainty is not ‘noise’ in the data that should be ignored, discarded, or removed by some post hoc coding procedure. Rather, it is a real phenomenon inherently part of fertility decision making” (Morgan 1981: 268). The 1965 NFS asked those respondents who indicated their intention to have more children, “Do you think you might later change your minds and decide not to have another child?” And it asked respondents who indicated the intention to stop, “Do you think you might later decide to have another child?” Learning that 7 percent did not know their intentions to begin with, 13 percent were uncertain of their intention to stop, and 50 percent were uncertain of their stated intention to have more, Morgan commented that these high levels of inconsistency between intentions and behaviors were “not surprising” (p. 280). Morgan's work pointed to flexibility as an inherent part of fertility intentions, worthy of further theoretical and empirical attention. But despite the fact that these two simple questions actually did, to some extent, index individuals’ willingness to revise their preferences, such questions are rarely used by researchers today and are seldom asked outside of the West.

Like Coombs, we believe that a structure underlies each person's stated ideal. However, rather than conceptualizing this structure numerically, as a type of statistical uncertainty, we focus on the level of flexibility that characterizes individual preferences. By flexibility we mean the extent to which preferences are designed to shift in the wake of the evolving contingencies. We posit that flexibility is measurable and intrinsically linked to fertility preferences and that measuring the prevalence of and variation in flexibility can increase understanding of fertility processes broadly. When viewing world fertility patterns from a fixed‐feasts perspective, an unacceptably large proportion of fertility preferences appear unstable, invalid, unpredictable, and untrustworthy; we argue here that for large portions of the world's population, this instability is not an anomaly to be corrected for, but, like movable feasts, an essential aspect of their nature.

## Data and methods

### Study context

The data for our study come from Tsogolo la Thanzi (TLT), a longitudinal study conducted in Balaka, Malawi designed to examine how, in the context of a generalized AIDS epidemic, young adults navigate the sometimes incompatible goals of enjoying sexual relationships, bearing children, and avoiding HIV infection. Balaka is a bustling township located in Malawi's southern region at the crossroads between a major road linking the country's political capital (Lilongwe) with its cultural capital (Zomba) and the rail route that ferries goods between Salima and Blantyre. The common refrain “In Balaka, every day is market day” attests to the vibrancy of this rapidly growing trading town. Several other pieces of information contextualize the setting in which we examine young adults’ fertility goals in the context of broader concerns.

First, the economic conditions characterizing Balaka are harsh. Despite the commercial activity, southern Malawi is poorer than the rest of the country. The southern region features lower levels of educational attainment and higher levels of poverty than the northern and central regions (MDHS 2010). Most residents of Balaka are subsistence farmers; there is just one paved road, and in 2009 only 12 percent of households had access to electricity. Second, of epidemiological relevance, the southern region has the country's most severe AIDS epidemic; in 2010, 15 percent of the population aged 15–49 in the southern region was infected with HIV, compared to 8 percent in the central and 7 percent in the northern region (ibid.). In pan‐African perspective, Balaka's epidemic might best be described as severe but improving: HIV prevalence in Malawi's southern region, estimated at 17 percent in 2004, has fallen to 12 percent but remains twice as high as prevalence in nearby regions. Recent causes for optimism include a decline in new infections, expanded access to antiretroviral treatment, and falling AIDS‐related mortality (ibid.). Despite these improvements, however, the epidemic has engendered widespread uncertainty: a sizable proportion of the population is unsure of their current HIV status, and worry about future infection is omnipresent for the vast majority of young adults (Kaler and Watkins 2010; Trinitapoli and Yeatman 2011; Watkins 2004). Third, the transition to adulthood unfolds quickly: women become sexually active around age 17, marry for the first time about a year later, and give birth to their first child about a year after that (Boileau et al. 2009; Clark, Poulin, and Kohler 2009; Poulin 2007).

The first wave of data collection for TLT took place between May and August 2009. A simple random sample of 1,505 female respondents was drawn from a sampling frame of 15 to 25 year olds living in villages within a seven‐kilometer radius of Balaka's main market. The catchment area includes a mix of rural and peri‐urban communities around the trading center.

TLT interviewers first contacted respondents in their homes and arranged a time for an interview. Respondents came to the research center, adjacent to the town's main market, and were interviewed in private rooms where their responses could not be overheard by family members and neighbors. Each survey took approximately 90 minutes. At Wave 1, refusal at the time of making an appointment and passive refusal by not showing up were rare (97 percent of sampled and eligible respondents completed a baseline interview). Eight waves of data were collected from this cohort of women through 2011, with survey rounds scheduled at four‐month intervals; the response rate at Wave 8 was 81 percent. In 2015, a ninth wave of data (TLT‐2) was collected from the original sample of respondents; TLT‐2 had an 80 percent response rate.

### Sample overview

The first panel of Table 1 describes all measures and summarizes the characteristics of the sample at Wave 1 (N = 1,505). At baseline, respondents ranged in age from 15 to 25, with a mean age of 19.5 years. Variability in rural/urban residence within the catchment area is captured by a standardized measure of distance from the town center. Mean education was 7.7 years—just shy of primary school completion. Half of respondents had ever been or were currently married. Thirteen percent of the sample was pregnant at baseline (not shown), and 10 percent of women reported having experienced a miscarriage or child death. Average parity was 0.79; about half the sample had no children, while others (N = 11) had four or five children at baseline. In contrast to van de Walle's (1992) respondents from Bamako nearly three decades ago, young women in Balaka had no trouble giving numeric responses to questions about ideal family size. Only two failed to respond to our question about IFS by giving a number. Ideal family size ranged from 0 to 8 children, with a mean of 3.22 children. By 2015 (six years later), 85.5 percent had at least one child (not shown), and mean parity was 1.92.

**Table 1:** TLT sample characteristics

	Wave 1, 2009				TLT‐2, 2015			
Variables	Mean	Std. dev.	Min	Max	Mean	Std. dev.	Min	Max
**Standard measure of fertility preferences**								
Ideal family size	3.22	1.09	0	8	3.53	0.99	1	8
**Flexibility**								
Total flexibility	12.34	8.44	0	36	11.19	11.31	0	36
Numeric flexibility	6.00	4.44	0	18	4.62	5.54	0	18
Timing flexibility	6.34	5.12	0	18	6.57	7.27	0	18
**Life‐course factors**								
Age	19.51	3.23	15	25	25.58	3.27	21	31
Ever married	0.50		0	1	0.84		0	1
Parity	0.79	0.96	0	5	1.92	1.30	0	6
**Socioeconomic status**								
Distance from town center (standardized)	−0.01	0.98	−1.27	4.33	—	—	—	—
Household goods	2.61	1.71	0	8	2.70	1.79	0	8
Years of education	7.66	2.81	0	14	8.22	3.18	0	15
**Calculus of conscious choice**								
Numeracy^a^	1.99	0.74	1	3	—	—	—	—
Children just happen	0.46		0	1	0.43			
Think about future	3.14	0.92	0	4	3.04	1.01	0	4
**Existential uncertainty**								
Miscarriage or child death	0.10		0	1	0.18		0	1
Probability of death within 1 year	1.93	2.44	0	10	3.27	2.59	0	10
Funerals attended in past month	1.21	1.32	0	20	1.17	1.16	0	10
**Fertility‐related behaviors**								
Using modern contraception	18.21				45.58			
	N = 1,505				N = 1,200			

a Measured at Wave 7.

NOTE: Education‐level standardized mean imputed for 283 missing cases.

SOURCE: Tsogolo la Thanzi, Wave 1 (2009) and TLT‐2 (2015).

### Measuring fertility preferences and flexibility

Our two key measures of fertility preferences are ideal family size (a numeric preference) and ideal time to next birth (a timing preference). To measure IFS, we asked: “People often do not have exactly the same number of children they want to have. If you could have exactly the number of children you want, how many children would you want to have?” Response categories for ideal time to next birth range from one (“as soon as possible”) to six (“five or more years”). Immediately following these questions, we endeavored to measure the flexibility of preferences. Interviewers asked each respondent how she would respond to each of 18 events and circumstances. Faced with scenarios that commonly occur in Malawi (food shortage, death of a parent, relationship instability), would her preference for the number of children she stated earlier increase, decrease, or stay the same? Would the event alter her desired timing (sooner, later, no change)? Questions in the flexibility module (see Appendix) were asked during the 2009 baseline survey and again in 2015.^1^

### Analytic approach

Our analysis proceeds in four parts. First, we use the baseline data from 2009 to describe the flexible nature of fertility preferences and to quantify the levels of flexibility both in individual women and in the study population along the dimensions of timing and number. Second, we examine the extent to which flexibility is patterned by life‐course stage and known markers of disadvantage, represents a mindset about fertility that lies outside the calculus of conscious choice, or is best understood as a response to uncertainty. We examine flexibility's relationship to:

- life‐course markers (age, marital status, parity);
- socio‐demographic factors including urban/rural residence, household wealth (following closely from the DHS household goods index—Rutstein and Johnson 2004), and educational attainment;
- the calculus of conscious choice, proxied in three ways: i) a direct measure of numeracy (wherein respondents were asked to solve simple math problems), ii) a single‐item measure of planning for the future (“How often, if at all, do you think about or plan for your future?” with Likert‐style responses), and iii) a binary indicator of agreement with the statement “You don't plan on having children, they just happen”; and
- measures designed to operationalize existential uncertainty. The first distinguishes women who report in their pregnancy and childbearing histories ever having experienced a miscarriage or the death of a child. The second gauges experience of death in the respondent's own network by indexing the number of funerals she attended in the past month. The third gauges each respondent's own sense of mortality using an interactive solicitation method in which respondents are given a pile of 10 beans and asked to shift from one plate to another the number of beans representing the likelihood a given event will occur within a specified time frame. Ten beans indicate absolute certainty the event will occur, zero beans absolute certainty it will not, and five beans a 50‐50 chance. We measure the perceived likelihood of imminent HIV infection using the prompt: “Pick the number of beans that reflects how likely it is that you will die within a 1‐year period beginning today.” This technique has been used successfully in a variety of cultural contexts to generate assessments of child mortality, HIV prevalence, food shortages, and adult mortality (Delavande, Giné, and McKenzie 2011; Delavande and Kohler 2009; Delavande and Rohwedder 2011; Trinitapoli and Yeatman 2011).

Third, we assess the extent to which relevant preferences, behaviors, and outcomes vary by level of flexibility. We start by estimating the relationship between flexibility at baseline and observed instability in IFS over the subsequent seven waves. We further test whether flexibility affects fertility‐related behaviors (use of modern contraception) and outcomes (pregnancy and surprise pregnancy).

Fourth, we re‐examine flexibility in this same cohort of women six years later. Combining a dynamic view of flexibility with a dynamic view of fertility preferences allows us to adjudicate between trait‐based explanations and perspectives on flexibility that view it as part of a developmental trajectory, responsive to context, and, perhaps, a more temporary state.

## Results

### Flexibility: Domains, dimensions, and levels

**Figure 1:**
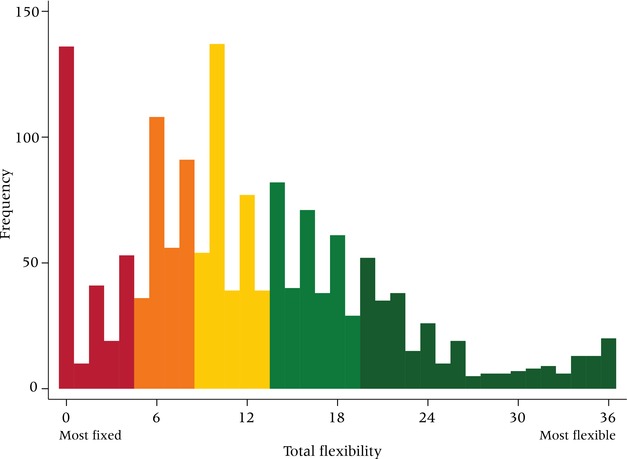
Level of flexibility (range 0–36) among women in Balaka, 2009 NOTE: Figure shows quintiles from most fixed to most flexible. N=1,505 women. SOURCE: Tsogolo la Thanzi, Wave 1

Table 2 presents detailed information on the diversity of conditions under which respondents indicated in 2009 whether and how they would adjust their fertility preferences. We categorized our conditions into three domains: AIDS‐related factors (the first group in the table), economic factors, and family factors.^2^ On average, respondents indicated movement in fertility preferences on six of the 18 conditions for both their desired number of children and the desired timing of pregnancies. While 10.5 percent of young women in Balaka reported no movement in their numeric preferences for any of the conditions, 3.5 percent anticipated a change for every one of the conditions presented to them (see Figure 1). With respect to timing preferences, 14.5 percent reported no movement, and 4.7 percent anticipated movement in response to every condition we inquired about.

**Table 2:** Descriptive overview of flexibility, 2009

	Numeric			Timing		
	Flexible		Fixed	Flexible		Fixed
Condition	More	Fewer	No change	Sooner	Later	No change
**All AIDS conditions**						
Partner starts losing weight and suspects AIDS	0.07	66.29	33.64	35.57	25.27	39.16
You start losing weight and suspect AIDS	0.00	66.05	33.95	34.91	26.38	38.71
You hear rumors your partner is unfaithful	0.13	54.86	45.01	24.45	30.51	45.04
**All economic conditions**						
Partner migrates to South Africa for work	1.46	40.20	58.34	13.29	34.09	52.62
Anticipated maize shortage	0.20	20.61	79.19	8.45	19.23	72.32
You secure a steady job	9.63	8.77	81.59	5.91	19.14	74.95
Husband secures a steady job	9.97	8.37	81.66	5.25	20.73	74.02
You win the lottery	6.32	8.78	84.91	6.12	16.02	77.86
Secondary school becomes free	5.85	7.44	86.71	5.25	16.61	78.14
Primary school uniforms and materials become free	4.78	8.04	87.18	4.32	17.54	78.14
**All family conditions**						
Partner wants fewer children	2.06	61.86	36.08	17.34	27.44	55.22
Sister dies and you foster her three children	3.65	49.57	46.78	14.15	31.89	53.95
Partner wants more children	36.17	6.98	56.85	8.65	31.47	59.88
You have only boy children	24.53	7.71	67.75	6.05	24.60	69.35
You have only girl children	23.26	7.44	69.30	6.18	22.74	71.08
Youngest child becomes ill	4.86	15.64	79.51	6.91	22.27	70.81
Mother dies	1.60	14.23	84.18	8.84	13.49	77.67
Mother becomes ill	1.26	10.76	87.97	5.58	16.41	78.01
N = 1,505						

NOTE: For each condition, respondents who indicated they would adjust their fertility preferences (i.e., more, fewer; sooner, later) are flexible on that condition; those indicating they would maintain their stated preference (i.e., no change) are considered fixed.

SOURCE: Tsogolo la Thanzi, Wave 1

For 13 conditions in Table 2, including all of the economic conditions (e.g., winning the lottery, new policies to make the education of children more affordable, a steep rise in the price of food) and most conditions related to family crises such as the illness or death of a parent, less than 50 percent of the sample reported that they would respond by changing their fertility preferences—either in number or timing. Our data provide no evidence of strong sex preferences of offspring, although more than one‐fifth of women expressed a clear desire for a mixed‐sex household by stating they would continue having more children if they had only boys or only girls.^3^

A few conditions do, however, appear to incite change for a majority of the TLT sample. Of the five conditions that elicit numeric and timing changes for the majority, four are AIDS‐related — two explicitly (suspecting AIDS for yourself or your partner due to weight loss) and two for which AIDS is strongly implied (hearing rumors of a partner's unfaithfulness and fostering children following a sibling's death). The other most consequential condition is one's partner wanting fewer children.^4^

With respect to timing preferences, AIDS‐related conditions are the only conditions that lead a sizable minority of respondents to say they would accelerate their childbearing; all other conditions tend to elicit delays. While 55 percent of women report that they would want fewer children if they heard rumors of an unfaithful partner, 25 percent say that they would have their children sooner—presumably as a strategy for maintaining the relationship or having children before becoming infected with HIV themselves (Hayford, Agadjanian, and Luz 2012; Trinitapoli and Yeatman 2011).^5^

To operationalize flexibility, we distinguished respondents who list “No change” (coded 0) from those who indicate a likely change (more/fewer *or* sooner/later, coded 1) for each item, without consideration to direction of change. For each respondent, we then summed responses to all 36 conditions to capture her level of flexibility in a simple additive scale. The analyses that follow center primarily on this scale, which ranges from 0 to 36; occasionally we employ alternate specifications, such as a measure examining numeric flexibility only (0–18) or a distilled measure in which the total flexibility score is converted into quintiles (as in Figure 1).

### Flexibility: Patterned

In 2009, over 90 percent of the TLT sample indicated some flexibility, with almost 20 percent expressing more than 20 likely changes in response to the 36 hypothetical conditions. This finding raises questions about the ways in which flexibility is patterned. Is flexibility level tied to life‐course steps—chronological and/or social age? Is it patterned socio‐demographically, along the same lines of disadvantage we observe for other fertility‐related outcomes? Does it indicate a pre‐transition mindset, in which fertility lies outside the calculus of conscious choice? Or is flexibility, as some have suggested, a strategic response to uncertainty?

**Figure 2:**
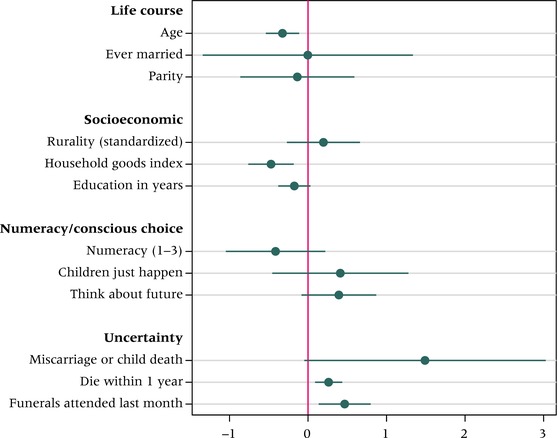
Predictors of flexibility among young women in Balaka, 2009 NOTE: Displays unstandardized coefficients and 95 percent confidence intervals based on a single OLS model. Numeracy measure comes from Wave 7. Listwise deletion to deal with three missing cases. N=1,502. SOURCE: Tsogolo la Thanzi, Wave 1.

Our approach to aligning proxy measures with each theoretical explanation for flexibility is captured by the organizational subheadings of Table 1. Figure 2 depicts the results from a single OLS model (Jann 2014), assessing 12 possible correlates of flexibility, where the outcome ranges from 0 to 36, as depicted in Figure 1. Alternate specifications, including a Poisson model and an ordered logit model of the flexibility quintiles, produced substantively identical conclusions. Considering three proxies for stage of the life course—age, marital status, and parity—we observe a significant, negative age gradient, suggesting that flexibility is most pronounced among the young. That we observe no differences in flexibility by marital status or parity, however, suggests that flexibility is not primarily a function of the life‐course trajectory. Evidence for a socioeconomic gradient is similarly mixed: flexibility is negatively associated with household wealth, but is not more prevalent among rural women and features no educational gradient.

The three proxies intended to operationalize numeracy and the calculus of conscious choice have no significant explanatory power for explaining variance in the levels of flexibility among young women in Balaka. Even though over 46 percent of TLT respondents agreed with the statement “You don't plan on having children; children just happen,” this attitudinal measure is unassociated with flexibility at baseline, and the same is true of our single‐item measure of future orientation.

It is among the three variables intended to measure existential uncertainty that we see strong and significant relationships with flexibility. Net of other variables, flexibility is higher among women who, prior to their interview in 2009, had ever experienced a miscarriage (N = 60) or the death of a child (N = 88). Additionally, perceived risk of mortality and number of funerals attended in the preceding month are strongly and positively associated with flexibility.

In short, Balaka's poorest young women, and the youngest, exhibit higher levels of flexibility than their wealthier and more mature counterparts. Admittedly, the calculus of conscious choice is a difficult concept to measure empirically; however, the fact that flexibility does not co‐vary with education, numeracy, or our attitudinal measures about human versus divine control over childbearing suggests that flexibility is not the result of a stalled pre‐transition mindset. Flexibility appears instead to be a response to existential uncertainty; that it tracks so closely with proximity to death (child death, deaths through funerals, and perceived mortality) resonates with findings like those of Bledsoe, Banja, and Hill (1998) on rural Gambia, Defo (1998) on Cameroon, and Sandberg (2005, 2006) on Nepal.

### Relevance of flexibility for fertility‐related preferences, behaviors, and outcomes

**Figure 3:**
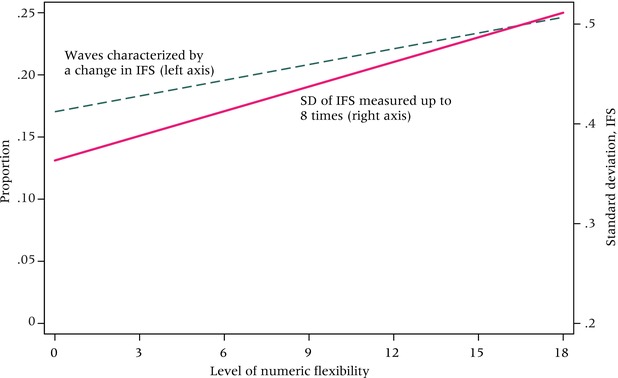
IFS changes over time by numeric flexibility level (0–18) at baseline N=1,505 SOURCE: Tsogolo la Thanzi, Waves 1–8.

The prospective data from TLT allow us to examine the relevance of flexibility for subsequent changes in IFS (i.e., preference instability) and for fertility‐related behaviors and outcomes. Figure 3 depicts the relationship between the measure of numeric flexibility (0–18) and the number of shifts in IFS women report over the course of the eight waves. The dashed line considers IFS instability proportionally: each woman's number of shifts is standardized by the total number of waves she contributed to the study. Shifts in IFS are fairly straightforward to measure.^6^ A woman who reported four children at Wave 1 and then three at Wave 2 is counted as having “changed” one time. If she persisted at three for the subsequent six waves, her proportion change is 0.125 (1/8); if she contributed only six waves to the study, 0.167 (1/6). Preference instability can also be captured by the standard deviation surrounding each woman's IFS over the eight waves, represented in Figure 3 by the solid line. Unlike the standardized count of changes, operationalizing observed flexibility with the standard deviation of her IFS distinguishes between a woman who answers 2‐2‐3‐5‐6 (3 changes) and another three‐change answer of 2‐3‐3‐2‐3.

Figure 3 shows that the level of numeric flexibility recorded at baseline is associated with both measures of IFS in/stability over the three‐year period. This lends support to the possibility that researchers can proxy flexibility prior to observing it, simply by asking.

While the analyses presented thus far have been limited to the domain of preferences, we believe that further investigation of flexibility will inform broader questions about how people anticipate, think about, and behave vis‐à‐vis childbearing. Here we offer preliminary evidence that flexibility is an integral part of fertility‐related behavior. Because women in the TLT sample are still young and have not yet completed their reproductive careers, and because flexibility itself is not always stable over time, establishing the extent to which flexibility may engender a particular and distinctive approach to fertility is both conceptually and statistically difficult. Below, we assess the relationship in the simplest manner by examining flexibility's relationship to surprise pregnancies and contraceptive use.

**Figure 4:**
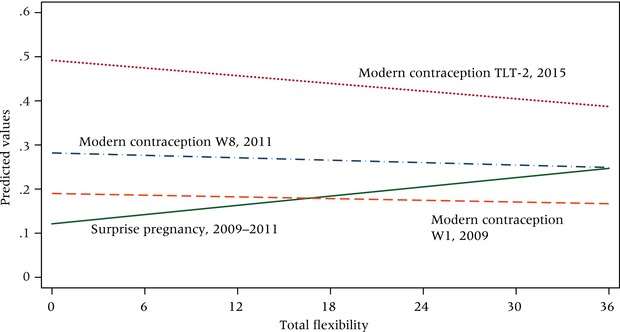
Surprise pregnancy and modern contraceptive use at three points in time by prospective flexibility score (range 0–36) NOTE: Based on predicted probabilities; models control for age, parity, marital status, distance from town center, household goods index, years of education, and stated desire to have a child within two years. Ns vary depending on outcome, ranging from 1,067 for surprise pregnancy to 1,505 for modern contraceptive use measured at W1. SOURCE: Tsogolo la Thanzi, Waves 1–8 and TLT‐2, 2015.

To identify pregnancies, and more specifically “surprise” pregnancies, we use data collected during Waves 2–8 (2009–2011). Surprise is gauged subjectively, and measurement is straightforward. At each survey wave after the baseline, women were asked whether they had learned, during the inter‐survey period, that they were pregnant. Women who answered yes were asked: “Was the pregnancy a surprise?” About 28 percent of women (N = 405) experienced at least one pregnancy, and more than half of these (N = 226, 15.5 percent) said that the pregnancy was a surprise. Figure 4 displays results from four separate logistic regression models: the solid line depicts the predicted probability of having experienced a surprise pregnancy (vs. not experiencing any pregnancy) by flexibility score. The underlying model controls for age, parity, marital status, education, household goods, and rural/urban residence—all measured at baseline and all operating as we would expect from the literature (not shown, available upon request). While flexibility level measured in 2009 has no relationship to the experience of pregnancy broadly (ancillary model, not shown), it is positively and statistically significantly related to having experienced a surprise pregnancy over the course of the study, with each point on the flexibility scale elevating by about 2 percent the odds of having been surprised (Coef. = .021, aOR 1.02).

We additionally assessed the relationship between flexibility and current use of modern contraception (IUD, injectable, implant, pill, or sterilization), measuring contraceptive use contemporaneously with the flexibility measure (2009), again at Wave 8 (2011), and finally during the 2015 follow‐up. As shown by the dotted and dashed lines in Figure 4, we observe no association between flexibility and the use of modern contraception in either 2009 or 2011, during which time contraceptive use remained low (18 and 27 percent, respectively). Net of basic sociodemographic controls and the desire to become pregnant in the near future, flexibility has a strong negative relationship with current use of a modern contraceptive in 2015, when current use stood at 46 percent. In other words, women who were the most flexible were the least likely to be using modern methods of contraception.

Taken together, these findings suggest that women who approach fertility flexibly during young adulthood behave differently from their counterparts who take a more fixed approach. Specifically, their fertility preferences change more over time, they are less likely to use modern contraception despite not articulating a desire to become pregnant, and they are more likely to experience surprise pregnancies.

### Flexibility revisited: TLT‐2, 2015 follow‐up

**Figure 5:**
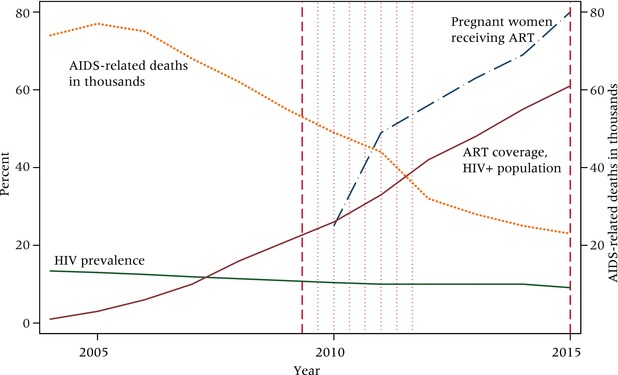
HIV and treatment context of Malawi, 2004–2015 NOTE: Vertical dashed lines represent time of flexibility module data collection; vertical dotted lines represent time of other survey rounds, specifically IFS, pregnancy, and contraceptive use. SOURCE: UNAIDS.

To this point we have examined the fertility preferences of a particular cohort of young women, but there are at least two reasons to expect flexibility itself to be dynamic. First, from a life‐course perspective, the “cloudy futures” approach to fertility suggests that intentions should resolve over time, as the fog of youth begins to dissipate and a referent future starts to become clear. That we observed an age gradient in flexibility in 2009 lends further support to this expectation and calls for a longitudinal examination of this issue. Second, although HIV prevalence was stable between 2009 and 2015, Malawi's epidemic changed considerably during these six years—particularly with respect to AIDS‐related mortality, thanks to improvements in access to lifesaving antiretroviral therapies (ART) distributed both through antenatal clinics where women are routinely tested for HIV and throughout the infected population of Balaka more widely. Figure 5 depicts these features of the epidemic for a 12‐year period, illustrating the precipitous decline in AIDS‐related mortality and improvements to ART coverage that characterized the first phase of the TLT study (2009–2011) and its immediate aftermath.

**Figure 6:**
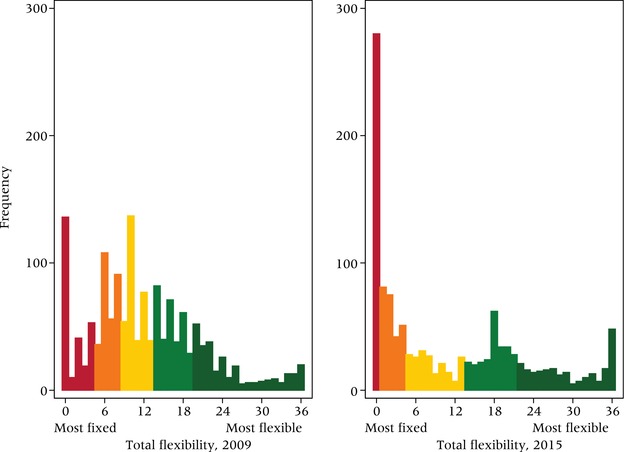
Levels of flexibility among young women in Balaka, 2009 and 2015 NOTE: Figure shows quintiles from most fixed to most flexible. N=1,200. SOURCE: Tsogolo la Thanzi.

In 2015, we repeated the flexibility module with the original TLT sample, now aged 21–31. Although the cohort had aged socially in expected ways (by mid‐2015, 84 percent had ever been married, and mean parity stood at just under 2 children), mean IFS (3.5) for the study population had not changed considerably, nor had the average levels of flexibility for either numeric (4.62) or timing (6.57) dimensions. (See Table 1.) Despite this apparent consistency, however, a closer look at the distributions and the components of flexibility six years later suggests that some important changes in flexibility are masked by the averages. First, Figure 6 depicts the distribution of flexibility among the women from whom we collected data at both points in time, revealing notable differences. Even though mean level of flexibility declines by only 1 point on the 36‐point scale, the number of women reporting zero flexibility more than doubled between 2009 and 2015. The distribution itself becomes slightly flatter, and variance nearly doubles (from 71.20 to 127.93).

Second, the full set of results from the follow‐up survey round, presented in Table 3, demonstrates that the components of flexibility in Balaka changed over time. The proportion of respondents who expected they would *not* change their preferences in response to each situation rose considerably: by 20 to 30 percentage points for the AIDS‐related conditions that invoked weight loss, almost 20 points for the unfaithfulness of a partner, and 15 points (numeric) for assuming additional fostering responsibilities. Compared to the portrait of flexibility we gathered in 2009, proportionally fewer TLT respondents believed themselves to be susceptible to their partners’ preferences either for more or for fewer children. At the same time, change became more common in a few of the categories: for example, a slightly larger proportion of respondents said their fertility preferences would be altered by the provision of primary school uniforms and materials. Examined in the aggregate, respondents’ fertility preferences may not have become decidedly less flexible over the study period, but the proportion of women who characterize their intentions as completely fixed grew from 9 to 23 percent, and AIDS‐related forces factor far less heavily into decision‐making in 2015 than they did in 2009.

**Table 3:** Descriptive overview of flexibility, 2015

	Numeric			Timing		
	Flexible		Fixed	Flexible		Fixed
Condition	More	Fewer	No change	Sooner	Later	No change
**All AIDS conditions**						
Partner starts losing weight and suspects AIDS	0.42	39.25	60.33	3.33	34.17	62.50
You start losing weight and suspect AIDS	0.25	40.83	58.92	3.83	35.42	60.75
You hear rumors your partner is unfaithful	0.50	34.50	65.00	2.92	34.92	62.17
**All economic conditions**						
Partner migrates to South Africa for work	0.83	30.50	68.67	3.17	39.08	57.75
Anticipated maize shortage	0.33	27.50	72.17	2.75	33.00	64.25
You secure a steady job	3.00	13.33	83.67	3.00	31.75	65.25
Husband secures a steady job	3.67	12.83	83.50	3.92	30.83	65.25
You win the lottery	2.67	13.67	83.67	3.67	31.92	64.42
Secondary school becomes free	4.00	12.50	83.50	4.00	31.08	64.92
Primary school uniforms and materials become free	4.25	13.92	81.83	4.33	30.33	65.33
**All family conditions**						
Partner wants fewer children	0.92	37.67	61.42	3.75	34.00	62.25
Sister dies and you foster her three children	3.25	35.42	61.33	3.75	38.58	57.67
Partner wants more children	9.92	12.67	77.42	3.25	34.67	62.08
You have only boy children	5.08	15.00	79.92	3.08	32.00	64.92
You have only girl children	5.42	13.17	81.42	3.00	31.08	65.92
Youngest child becomes ill	1.42	22.00	76.58	2.92	32.67	64.42
Mother dies	1.67	19.00	79.33	2.42	31.58	66.00
Mother becomes ill	1.33	19.33	79.33	2.50	30.17	67.33
N = 1,200						

NOTE: For each condition, respondents who indicated they would adjust their fertility preferences (i.e., more, fewer; sooner, later) are flexible on that condition; those indicating they would maintain their stated preference (i.e., no change) are considered fixed.

SOURCE: Tsogolo la Thanzi‐2, 2015.

**Figure 7:**
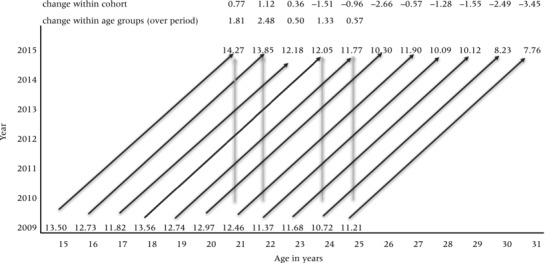
Age‐standardized view of flexibility level using a modified Lexis diagram NOTE: The Lexis diagram summarizes change in the average flexibility score (range 0–36) between 2009 and 2015 for each single‐year birth cohort. Comparisons can be made for each cohort over time (diagonal lines) or for age groups (e.g., 21‐year‐olds) at the two points in time (vertical lines). For example, the average flexibility level among 21‐year‐olds rose from 12.46 in 2009 to 14.27 in 2015 (change of 2.48). Among respondents who were 25 in 2009, flexiblity levels averaged 11.21 but dropped to 7.76 by 2015 (change of –3.45). SOURCE: Tsogolo la Thanzi, Wave 1 (2009) and TLT‐2 (2015).

Third, an age‐standardized view of flexibility level between the two time periods lends partial support to the theory that fertility preferences should crystalize, and flexibility decline, as the life course progresses. Figure 7 provides such a view using a modified Lexis diagram. Age is represented on the horizontal axis, time on the vertical axis. Comparisons between the two periods at each age can be made by reading from bottom to top, and changes within each one‐year cohort over the six‐year period can be gleaned by following the diagonal. Again, here, important patterns masked by the averages are evident from the Lexis view. For the ages (21–25) at which we can observe flexibility levels at both points in time, evidence that flexibility is declining is weak; indeed the average flexibility level characterizing each age group actually increases slightly during the inter‐survey period. The within‐cohort perspective, however, provides evidence that flexibility *declines* with age. The decline is most notable among the oldest respondents (30 and 31), and it is only among the youngest cohorts (15 and 16 at the outset of the study) that fertility preferences do not seem to solidify at all.

## Discussion

Our evidence from Balaka demonstrates that the flexibility accompanying fertility preferences is prevalent, patterned, and consequential. The questions we asked about circumstances that might lead women to change their stated ideal number and timing of children revealed that only a minority approaches their fertility preferences from a fixed perspective. Women's preferences are flexible enough to accommodate several types of uncertainty, including child mortality, relationship instability, precarious economic circumstances, and AIDS. Flexibility levels are not evenly distributed across the study population but are patterned by age, socioeconomic status, and—most demonstrably—existential uncertainty. This flexibility predicted subsequent instability in fertility preferences, contraceptive use (though not consistently), and surprise pregnancy. Finally, although flexibility declined modestly over the life course, flexibility levels among this same cohort six years later remain high.

While flexibility dominates in Balaka, it is not universal. In 2009 about 9 percent of women aged 15–25 exhibited no flexibility; that proportion more than doubled (to 23 percent) over the six‐year interval. Depending on the threshold we use to define low, somewhere between 8 and 11 percent of women exhibited very low levels of flexibility at both points in time—a fact that can be read as consistent with the emergence of a family‐limitation mentality pointed out by Casterline and Agyei‐Mensah (2017). A decline in flexibility can also be read from the Malawi Demographic and Health Surveys, which have documented an increase in sterilization from just over 10 percent of married women above age 40 in 2000 to more than 30 percent in 2015/16. Clearly, some Malawian women become fixed in their orientations at some point in their lives, but we maintain that a majority of young women remain flexible.

Across contexts, numeric and timing preferences are susceptible to change; this is particularly true of young adults facing an uncertain future. The concept of flexibility enables us to expect some of this change while retaining the meaning fertility researchers rightly attach to preferences, and it allows us to distinguish preference instability rooted in strategic flexibility from mere noise. From our view, flexibility is most productively understood as an approach to fertility that is schematic in nature. Following Johnson‐Hanks and colleagues (2011: 3), we emphasize the following characteristics of schematic phenomena—they tend to be underspecified, underdetermined, learned experientially rather than through direct instruction, and unobservable. The longitudinal view confirms that as a schematic approach, flexibility is not an individual‐level trait but a perspective that may vary with age, socioeconomic status, and existential uncertainty. Like other schemas, strategic flexibility—and its counterpart, fixedness—is the product of an accumulation of social interactions, linking to a variety of related schemas such as “what is a family”? or “how likely is it that my existing children will survive?” In all likelihood, no woman in Balaka has ever been instructed to make few concrete plans with respect to her childbearing. However, it is not difficult to imagine how a flexible orientation would get inculcated through the lessons transmitted with respect to planting and harvesting among subsistence farmers, through saving and selling in an economy of volatile currency, and through a heightened awareness of the fragility of life through the burial of many friends and kin.

In contrast to an earlier generation of research that distinguished between numeric and non‐numeric approaches to fertility, we find that flexibility can and does co‐exist with numeracy generally and with numeracy about children specifically. The language of family size is widely spoken across Malawi, but numeric fertility preferences are accompanied by very high levels of flexibility, suggesting that a single number here may not be as central to the calculus as it is in other parts of the world (Johnson‐Hanks 2007). For decades, researchers have remarked on the distinctiveness of the African fertility regime and the unique characteristics of its transition; flexibility may have special relevance for understanding the persistence of high fertility in sub‐Saharan Africa. For example, our insights from Balaka fit with arguments for the centrality of fertility postponement in sub‐Saharan Africa, where birth intervals are lengthening across the region (Moultrie et al. 2012; Timæus and Moultrie 2008). Flexibility helps make sense of an approach to fertility that is not based simply on age or parity but rather is a strategic response to life's contingencies. By engaging this dimension of fertility preferences directly, we see quite clearly that flexible women are not failing to plan but are planning differently.

Lest we imply that Malawian women or Africans generally are exceptional in having very flexible orientations to fertility, other researchers have classified responses to fertility questions as indicating uncertainty about preferences, which we see as indicating an underlying flexibility that may tend toward something universal. Flexibility has never been a mainstream concept in the demographic literature, but it does appear episodically. Nearly 40 years after Morgan's (1981) analysis of the preferences of American women in the 1960s and 1970s, evidence of flexibility among American women persists. Forty‐two percent of partnered women in the United States who gave a clear answer to the question about their intention to have (or not have) another child then stated that they were only somewhat or not at all sure about that intention (ICPSR n.d.). Depending on the precise definition used, between 31 percent and 47 percent of British women of reproductive age suggest that they are flexible in their preferences (Ní Bhrolcháin and Beaujouan 2011). Recent work on fertility preferences across Europe identifies a similar set of concepts, differentiating between distinct types of uncertainty and linking these to a new typology of fertility intentions, which includes contingent (flexible) and ambivalent (oscillating) outlooks in addition to pronatalist, antinatalist, and indifferent intentions (Bernardi, Mynarska, and Rossier 2015; Philipov and Bernardi 2012).

Flexibility need not always act as a prop to fertility. Bongaarts (2001) and others attributed the mismatch between fertility and preferences in low‐fertility contexts to “other constraints” or competing preferences, but they could also reflect an underlying flexibility that leads to lower fertility. In societies like Balaka, the most prevalent competing preferences (e.g., the desire to solidify a relationship (Yeatman and Trinitapoli 2013) or achieve a partner's higher preferences (Voas 2003)) encourage childbearing, helping to explain why flexibility may be one of the invisible forces sustaining high fertility in Malawi. But in parts of the world where constraints take the form of tight housing markets, rising health care costs, and labor market instability, the consequences of flexibility may manifest themselves in the opposite direction.

The highly contextualized, intensive, longitudinal design of the TLT study allowed us to explore flexibility in a detailed and tailored way that engaged local realities, uncertainties, constraints, and concerns. At the same time, this approach has limitations. Data collection efforts of this sort are expensive and time‐consuming. Furthermore, this kind of context‐specific, embedded approach to measuring flexibility is difficult to scale up. The ability to make clear, cross‐cultural comparisons will be essential for advancing research on flexibility with respect to its role in fertility transitions, but standardized measures cannot retain the contextual and temporal specificity we demanded here. As a simple next step, the development of even a few abbreviated measures of flexibility could go a long way toward implementing a movable‐feast approach within the study of fertility.

## Notes

1 Appendix is available at the supporting information tab at wileyonlinelibrary.com/journal/pdr. Our approach to measuring flexibility reflects an accumulation of influences from the fertility literature over the past 50 years. The following are some specific studies that were most influential at the design stage: sex preference (Coombs, Coombs, and McClelland 1975; Knodel et al. 1996), extended family props to fertility (Lesthaeghe 1980), the cost of schooling (Caldwell 1980), the importance of spacing vs. stopping in African societies (Kirk and Pillet 1998), men's preferences in Africa (Bankole 1995; Dodoo 1998), HIV and AIDS (Gregson 1994; Yeatman 2009), relationship formation and instability (Bongaarts 1978; Bongaarts, Frank, and Lesthaeghe 1984; Davis and Blake 1956), and labor market factors (Becker 1960; Easterlin 1975).

2 Initially, these categories were made based on face‐validity alone. However, we tested all groupings empirically: Cronbach's alpha for the disaggregated flexibility scales was >.73 for all six.

3 These two measures are highly correlated at .86.

4 Analyses with TLT data show that both men and women adjust their preferences in the direction of their partner's preferences (Yeatman and Sennott 2014).

5 Other analyses using TLT data have shown that women's numeric and timing preferences changed over time in patterned ways, most predictably by relationship changes and economic factors (Sennott and Yeatman 2012; Yeatman et al. 2013).

6 Because time to next birth references *next* birth, it needs to be calibrated to take into account the passage of time and new pregnancies. Thus, changes in timing preferences cannot be validated in an analogous way.

## References

[padr12114-bib-0001] Agadjanian, Victor . 2005 “Fraught with ambivalence: Reproductive intentions and contraceptive choices in a sub‐Saharan fertility transition,” Population Research and Policy Review 24(6): 617–645.

[padr12114-bib-0002] Bachan, Lauren . 2015 “Safety nets and social reproduction: Three essays on child fostering in sub‐Saharan Africa in the era of AIDS,” Ph.D. dissertation, Pennsylvania State University, State College, PA.

[padr12114-bib-0003] Bankole, Akinrinola . 1995 “Desired fertility and fertility behaviour among the Yoruba of Nigeria: A study of couple preferences and subsequent fertility,” Population Studies 49(2): 317–328.

[padr12114-bib-0004] Bankole, Akinrinola and Alex Chika Ezeh . 1999 “Unmet need for couples: An analytical framework and evaluation with DHS data,” Population Research and Policy Review 18(6): 579–605.

[padr12114-bib-0005] Bankole, Akinrinola and Charles F. Westoff . 1995 Childbearing Attitudes and Intentions. Macro International, Inc., http://dhsprogram.com/publications/publication-cs17-comparative-reports.cfm. Accessed January 28, 2017

[padr12114-bib-0006] Becker, Gary S. 1960 “An economic analysis of fertility,” in Universities‐National Bureau of Economic Research (ed.), Demographic and Economic Change in Developed Countries. New York: Columbia University Press and National Bureau of Economic Research, pp. 209–231.

[padr12114-bib-0007] Bernardi, Laura , Monika Mynarska , and Clémentine Rossier . 2015 “Uncertain, changing and situated fertility intentions,” in Philipov D., Liefbroer A C., and Klobas J. (eds.), Reproductive Decision‐Making in a Macro‐Micro Perspective. Dordrecht: Springer Netherlands, pp. 113–139.

[padr12114-bib-0008] Berry, Sara S. 1993 No Condition Is Permanent: The Social Dynamics of Agrarian Change in sub‐Saharan Africa. Madison: University of Wisconsin Press.

[padr12114-bib-0009] Bledsoe, Caroline , Fatoumatta Banja , and Allan G. Hill . 1998 “Reproductive mishaps and Western contraception: An African challenge to fertility theory,” Population and Development Review 24(1): 15–57.

[padr12114-bib-0010] Boileau, Catherine et al. 2009 “Sexual and marital trajectories and HIV infection among ever‐married women in rural Malawi,” Sexually Transmitted Infections 85(S1): i27–33. 19307337 10.1136/sti.2008.033969 PMC2654116

[padr12114-bib-0011] Bongaarts, John . 1978 “A framework for analyzing the proximate determinants of fertility,” Population and Development Review 4(1): 105–132.

[padr12114-bib-0012] Bongaarts, John . 1991 “The KAP‐gap and the unmet need for contraception,” Population and Development Review 17(2): 293–313.

[padr12114-bib-0013] Bongaarts, John . 2006 “The causes of stalling fertility transitions,” Studies in Family Planning 37(1): 1–16. 16570726 10.1111/j.1728-4465.2006.00079.x

[padr12114-bib-0014] Bongaarts, John . 2017 “Africa's unique fertility transition,” Population and Development Review 43(Suppl.): 39–58. 10.1111/j.1728-4457.2013.00557.x PMC4011385 24812439

[padr12114-bib-0015] Bongaarts, John and John Casterline . 2013 “Fertility transition: is sub‐Saharan Africa different?,” Population and Development Review 38(Suppl.): 153–168. 24812439 10.1111/j.1728-4457.2013.00557.x PMC4011385

[padr12114-bib-0016] Bongaarts, John , Odile Frank , and Ron Lesthaeghe . 1984 “The proximate determinants of fertility in sub‐Saharan Africa,” Population and Development Review 10(3): 511–537.

[padr12114-bib-0017] Butt, Bilal . 2011 “Coping with uncertainty and variability: The influence of protected areas on pastoral herding strategies in East Africa,” Human Ecology 39(3): 289.

[padr12114-bib-0018] Cain, Mead . 1981 “Risk and insurance: Perspectives on fertility and agrarian change in India and Bangladesh,” Population and Development Review 7(3): 435–474.

[padr12114-bib-0019] Caldwell, John C. 1980 “Mass education as a determinant of the timing of fertility decline,” Population and Development Review 6(2): 225–255.

[padr12114-bib-0020] Caldwell, John C. and Pat Caldwell . 2002 “Africa: The new family planning frontier,” Studies in Family Planning 33(1): 76–86. 11974421 10.1111/j.1728-4465.2002.00076.x

[padr12114-bib-0021] Caldwell, John C. , I. O. Orubuloye , and Pat Caldwell . 1992 “Fertility decline in Africa: A new type of transition?,” Population and Development Review 18(2): 211–242.

[padr12114-bib-0022] Casterline, John B. and Samuel Agyei‐Mensah . 2017 “Fertility desires and the course of fertility decline in sub‐Saharan Africa,” Population and Development Review 43(Suppl.): 84–111.

[padr12114-bib-0023] Casterline, John B. and Laila O. El‐Zeini . 2007 “The estimation of unwanted fertility,” Demography 44(4): 729–745. 18232208 10.1353/dem.2007.0043

[padr12114-bib-0024] Casterline, John and Siqi Han . 2017 “Unrealized fertility: Fertility desires at the end of the reproductive career,” Demographic Research 36(14): 427–454.

[padr12114-bib-0025] Clark, Shelley , Michelle Poulin , and Hans‐Peter Kohler . 2009 “Marital aspirations, sexual behaviors, and HIV/AIDS in rural Malawi,” Journal of Marriage and the Family 71(2): 396–416. 20161389 10.1111/j.1741-3737.2009.00607.x PMC2782839

[padr12114-bib-0026] Coale, Ansley J. 1973 “The demographic transition reconsidered,” in Proceedings of the International Population Conference, Liège. Liège, Belgium: International Union for the Scientific Study of Population, pp. 53–57.

[padr12114-bib-0027] Coombs, Clyde H. , Lolagene C. Coombs , and Gary H. McClelland . 1975 “Preference scales for number and sex of children,” Population Studies 29(2): 273–298.

[padr12114-bib-0028] Coombs, Lolagene C. 1974 “The measurement of family size preferences and subsequent fertility,” Demography 11(4): 587–611. 21279747 10.2307/2060472

[padr12114-bib-0029] Dare, O.O. and J.G. Cleland . 1994 “Reliability and validity of survey data on sexual behavior,” Health Transition Review 4(Suppl.): 93–110. 10150527

[padr12114-bib-0030] Davis, Kingsley and Judith Blake . 1956 “Social structure and fertility: An analytic framework,” Economic Development and Cultural Change 4(3): 211–235.

[padr12114-bib-0031] Defo, Barthélémy Kuate . 1998 “Fertility response to infant and child mortality in Africa with special reference to Cameroon,” in Montgomery Mark R. and Cohen Barney (eds.), From Death to Birth: Mortality Decline and Reproductive Change. Washington, DC: National Academies Press, pp. 254–315. 25121235

[padr12114-bib-0032] Delavande, Adeline , Xavier Giné , and David McKenzie . 2011 “Measuring subjective expectations in developing countries: A critical review and new evidence,” Journal of Development Economics 94(2): 151–163.

[padr12114-bib-0033] Delavande, Adeline and Hans‐Peter Kohler . 2009 “Subjective expectations in the context of HIV/AIDS in Malawi,” Demographic Research 20(31): 817–874. 19946378 10.4054/DemRes.2009.20.31 PMC2784667

[padr12114-bib-0034] Delavande, Adeline and Susann Rohwedder . 2011 “Differential survival in Europe and the United States: Estimates based on subjective probabilities of survival,” Demography 48(4): 1377–1400. 22042664 10.1007/s13524-011-0066-8 PMC3609718

[padr12114-bib-0035] Dodoo, Francis . 1998 “Men matter: Additive and interactive gendered preferences and reproductive behavior in Kenya,” Demography 35(2): 229–242. 9622784

[padr12114-bib-0036] Easterlin, Richard A. 1975 “Economic framework for fertility analysis,” Studies in Family Planning 6(3): 54–63. 1118873

[padr12114-bib-0037] Frank, Odile and John Bongaarts . 1991 “Behavioral and biological determinants of fertility transition in sub‐Saharan Africa,” Statistics in Medicine 10(2): 161–175. 2052797 10.1002/sim.4780100203

[padr12114-bib-0038] Frye, Margaret and Lauren Bachan . 2017 “The demography of words: The global decline in non‐numeric fertility preferences, 1993–2011,” Population Studies 71(2): 187–209. 28440109 10.1080/00324728.2017.1304565 PMC5525551

[padr12114-bib-0039] Gregson, Simon . 1994 “Will HIV become a major determinant of fertility in sub‐Saharan Africa?,” Journal of Development Studies 30(3): 650–679.

[padr12114-bib-0040] Günther, Isabel and Kenneth Harttgen . 2016 “Desired fertility and number of children born across time and space,” Demography 53(1): 55–83. 26786205 10.1007/s13524-015-0451-9

[padr12114-bib-0041] Hayford, Sarah R. 2009 “The evolution of fertility expectations over the life course,” Demography 46(4): 765–783. 20084828 10.1353/dem.0.0073 PMC2831352

[padr12114-bib-0042] Hayford, Sarah R. , Victor Agadjanian , and Luciana Luz . 2012 “Now or never: Perceived HIV status and fertility intentions in rural Mozambique,” Studies in Family Planning 43(3): 191–199. 23185862 10.1111/j.1728-4465.2012.00317.x PMC3662548

[padr12114-bib-0043] Heiland, Frank , Alexia Prskawetz , and Warren C. Sanderson . 2008 “Are individuals’ desired family sizes stable? Evidence from West German panel data,” European Journal of Population 24(2): 129.

[padr12114-bib-0044] Iacovou, Maria and LaraPatrício Tavares . 2011 “Yearning, learning, and conceding: Reasons men and women change their childbearing intentions,” Population and Development Review 37(1): 89–123. 21735613 10.1111/j.1728-4457.2011.00391.x

[padr12114-bib-0045] ICPSR . n.d. “National Survey of Family Growth, 2013–2015,” http://www.icpsr.umich.edu/icpsradmin/nsfg/variable/818136?vg=10324&studyNumber=9999. Accessed February 23, 2017.

[padr12114-bib-0046] Jann, Benn . 2014 “Plotting regression coefficients and other estimates,” Stata Journal 14(4): 708–737.

[padr12114-bib-0047] Johnson‐Hanks, Jennifer . 2005 “When the future decides—uncertainty and intentional action in contemporary Cameroon,” Current Anthropology 46(3): 363–385.

[padr12114-bib-0048] Johnson‐Hanks, Jennifer . 2007 “Natural intentions: Fertility decline in the African Demographic and Health Surveys,” American Journal of Sociology 112(4): 1008–1043.

[padr12114-bib-0049] Johnson‐Hanks, Jennifer A. et al. 2011 Understanding Family Change and Variation: Toward a Theory of Conjunctural Action. New York: Springer.

[padr12114-bib-0050] Kaler, Amy and Susan Watkins . 2010 “Asking God about the date you will die: HIV testing as a zone of uncertainty in rural Malawi,” Demographic Research 23(32): 905–932. 21562614 10.4054/DemRes.2010.23.32 PMC3090147

[padr12114-bib-0051] Kirk, Dudley and Bernard Pillet . 1998 “Fertility levels, trends, and differentials in sub‐Saharan Africa in the 1980s and 1990s,” Studies in Family Planning 29(1): 1–22. 9561666

[padr12114-bib-0052] Knodel, John , Vipan Prachuabmoh Ruffolo , Pakamas Ratanalangkarn , and Kua Wongboonsin . 1996 “Reproductive preferences and fertility trends in post‐transition Thailand,” Studies in Family Planning 27(6): 307–318. 8986029

[padr12114-bib-0053] Kodzi, Ivy , David Johnson , and John B. Casterline . 2010 “Examining the predictive value of fertility preferences among Ghanaian women,” Demographic Research 22(30): 965–984. 23970826 10.4054/DemRes.2010.22.30 PMC3747569

[padr12114-bib-0054] Lee, R. D. 1980 “Aiming at a moving target: Period fertility and changing reproductive goals,” Population Studies 34(2): 205–226. 22077121 10.1080/00324728.1980.10410385

[padr12114-bib-0055] LeGrand, Thomas , Todd Koppenhaver , Nathalie Mondain , and Sara Randall . 2003 “Reassessing the insurance effect: A qualitative analysis of fertility behavior in Senegal and Zimbabwe,” Population and Development Review 29(3): 375–403.

[padr12114-bib-0056] Lesthaeghe, Ron . 1980 “On the social control of human reproduction,” Population and Development Review 6(4): 527–548.

[padr12114-bib-0058] Lev, Larry and David J. Campbell . 1987 “The temporal dimension in farming systems research: The importance of maintaining flexibility under conditions of uncertainty,” Journal of Rural Studies 3(2): 123–132.

[padr12114-bib-0059] Liefbroer, Aart C. 2009 “Changes in family size intentions across young adulthood: A life‐course perspective,” European Journal of Population 25(4): 363–386. 20016795 10.1007/s10680-008-9173-7 PMC2791833

[padr12114-bib-0060] Mbacké, Cheikh . 1994 “Family planning programs and fertility transition in sub‐Saharan Africa: Review of *Population Dynamics of Sub‐Saharan Africa* ,” Population and Development Review 20(1): 188–193.

[padr12114-bib-0061] MDHS . 2010 *Malawi Demographic and Health Survey* . http://dhsprogram.com/publications/publication-fr247-dhs-final-reports.cfm. Accessed June 25, 2011

[padr12114-bib-0062] Morgan, S. Philip . 1981 “Intention and uncertainty at later stages of childbearing: The United States 1965 and 1970,” Demography 18(3): 267–285. 7262367

[padr12114-bib-0063] Moultrie, Tom A. , Takudzwa S. Sayi , and Ian M. Timæus . 2012 “Birth intervals, postponement, and fertility decline in Africa: A new type of transition?,” Population Studies 66(3): 241–258. 22891624 10.1080/00324728.2012.701660

[padr12114-bib-0064] Ní Bhrolcháin, Máire and Éva Beaujouan . 2011 “Uncertainty in fertility intentions in Britain, 1979–2007,” Vienna Yearbook of Population Research 9: 99–129.

[padr12114-bib-0065] Philipov, Dimiter and Laura Bernardi . 2012 “Concepts and operationalisation of reproductive decisions implementation in Austria, Germany and Switzerland,” Comparative Population Studies 36(2‐3): 495–530.

[padr12114-bib-0066] Poulin, Michelle J. 2007 “Sex, money, and premarital relationships in southern Malawi,” Social Science & Medicine 65(11): 2383–2393. 17764797 10.1016/j.socscimed.2007.05.030 PMC2758488

[padr12114-bib-0067] Pritchett, Lant H. 1994 “Desired fertility and the impact of population policies,” Population and Development Review 20(1): 1–55.

[padr12114-bib-0068] Randall, Sara and Thomas LeGrand . 2003 “Reproductive strategies and decisions in Senegal: The role of child mortality,” Population 58(6): 773–805.

[padr12114-bib-0069] Rutstein, Shea O. and Kiersten Johnson . 2004 “The DHS Wealth Index,” http://www.dhsprogram.com/publications/publication-cr6-comparative-reports.cfm). Accessed February 20, 2009.

[padr12114-bib-0070] Ryder, Norman B. 1973 “A critique of the National Fertility Study,” Demography 10(4): 495–506. 4804732

[padr12114-bib-0071] Sandberg, John . 2005 “The Influence of network mortality experience on nonnumeric response concerning expected family size: Evidence from a Nepalese mountain village,” Demography 42(4): 737–756. 16463919 10.1353/dem.2005.0035

[padr12114-bib-0072] Sandberg, John . 2006 “Infant mortality, social networks, and subsequent fertility,” American Sociological Review 71(2): 288–309.

[padr12114-bib-0073] Sedgh, Gilda and Rubina Hussain . 2014 “Reasons for contraceptive nonuse among women having unmet need for contraception in developing countries,” Studies in Family Planning 45(2): 151–169. 24931073 10.1111/j.1728-4465.2014.00382.x

[padr12114-bib-0074] Sennott, Christie and Sara Yeatman . 2012 “Stability and change in fertility preferences among young women in Malawi,” International Perspectives on Sexual and Reproductive Health 38(1): 34–42. 22481147 10.1363/3803412 PMC3322634

[padr12114-bib-0075] Shapiro, David, D. and Tesfayi Gebreselassie . 2008 “Fertility transition in sub‐Saharan Africa: Falling and stalling,” African Population Studies 23(1): 3–23.

[padr12114-bib-0076] Smith, Daniel Jordan . 2004 “Contradictions in Nigeria's fertility transition: The burdens and benefits of having people,” Population and Development Review 30(2): 221–238.

[padr12114-bib-0077] Timæus, Ian M. and Tom A. Moultrie . 2008 “On postponement and birth intervals,” Population and Development Review 34(3): 483–510.

[padr12114-bib-0078] Trinitapoli, Jenny and Sara E. Yeatman . 2011 “Uncertainty and fertility in a generalized AIDS epidemic,” American Sociological Review 76(6): 935–954. 22536003 10.1177/0003122411427672 PMC3334314

[padr12114-bib-0079] Udry, J. Richard . 1983 “Do couples make fertility plans one birth at a time?,” Demography 20(2): 117–128. 6862057

[padr12114-bib-0080] Verheijen, Janneke . 2013 “Balancing men, morals and money,” Ph.D. dissertation, Leiden, Amsterdam, Netherlands.

[padr12114-bib-0081] van de Walle, Etienne . 1992 “Fertility transition, conscious choice, and numeracy,” Demography 29(4): 487–502. 1483538

[padr12114-bib-0082] Watkins, Susan Cotts . 2004 “Navigating the AIDS epidemic in rural Malawi,” Population and Development Review 30(4): 673–705.

[padr12114-bib-0083] Yeatman, Sara E. 2009 “HIV infection and fertility preferences in rural Malawi,” Studies in Family Planning 40(4): 261–276. 21151844 10.1111/j.1728-4465.2009.00210.x PMC2998897

[padr12114-bib-0084] Yeatman, Sara and Christie Sennott . 2014 “The relationship between partners’ family‐size preferences in southern Malawi,” Studies in Family Planning 45(3): 361–377. 25207497 10.1111/j.1728-4465.2014.00396.x PMC4390032

[padr12114-bib-0085] Yeatman, Sara , Christie Sennott , and Steven Culpepper . 2013 “Young women's dynamic family size preferences in the context of transitioning fertility,” Demography 50(5): 1715–1737. 23619999 10.1007/s13524-013-0214-4 PMC3786023

[padr12114-bib-0086] Yeatman, Sara and Jenny Trinitapoli . 2013 “‘I will give birth but not too much’: HIV‐positive childbearing in rural Malawi,” in Liamputtong P. (ed.), Women, Motherhood and Living with HIV/AIDS. Dordrecht: Springer Netherlands pp. 93–109.

